# A single strain of *Bacteroides fragilis* protects gut integrity and reduces GVHD

**DOI:** 10.1172/jci.insight.136841

**Published:** 2021-02-08

**Authors:** M. Hanief Sofi, Yongxia Wu, Taylor Ticer, Steven Schutt, David Bastian, Hee-Jin Choi, Linlu Tian, Corey Mealer, Chen Liu, Caroline Westwater, Kent E. Armeson, Alexander V. Alekseyenko, Xue-Zhong Yu

**Affiliations:** 1 Department of Microbiology and Immunology, Hollings Cancer Center, College of Medicine, Medical University of South Carolina, Charleston, South Carolina, USA.; 2 Department of Pathology, Yale School of Medicine, New Haven, Connecticut, USA.; 3 Department of Oral Health Sciences, College of Dental Medicine, Medical University of South Carolina, Charleston, South Carolina, USA.; 4 Biomedical Informatics Center and Department of Public Health Sciences, College of Medicine, and Department of Healthcare Leadership & Management, College of Public Health Sciences, Medical University of South Carolina, Charleston, South Carolina, USA.

**Keywords:** Immunology, Transplantation, Adaptive immunity, Bone marrow transplantation, Cancer immunotherapy

## Abstract

Graft-versus-host disease (GVHD) is a pathological process caused by an exaggerated donor lymphocyte response to host antigens after allogeneic hematopoietic cell transplantation (allo-HCT). Donor T cells undergo extensive clonal expansion and differentiation, which culminate in damage to recipient target organs. Damage to the gastrointestinal tract is a main contributor to morbidity and mortality. The loss of diversity among intestinal bacteria caused by pretransplant conditioning regimens leads to an outgrowth of opportunistic pathogens and exacerbated GVHD after allo-HCT. Using murine models of allo-HCT, we found that an increase of *Bacteroides* in the intestinal microbiota of the recipients was associated with reduced GVHD in mice given fecal microbial transplantation. Administration of *Bacteroides fragilis* through oral gavage increased gut microbiota diversity and beneficial commensal bacteria and significantly ameliorated acute and chronic GVHD development. Preservation of gut integrity following *B*. *fragilis* exposure was likely attributed to increased short chain fatty acids, IL-22, and regulatory T cells, which in turn improved gut tight junction integrity and reduced inflammatory cytokine production of pathogenic T cells. The current study provides a proof of concept that a single strain of commensal bacteria can be a safe and effective means to protect gut integrity and ameliorate GVHD after allo-HCT.

## Graphical abstract

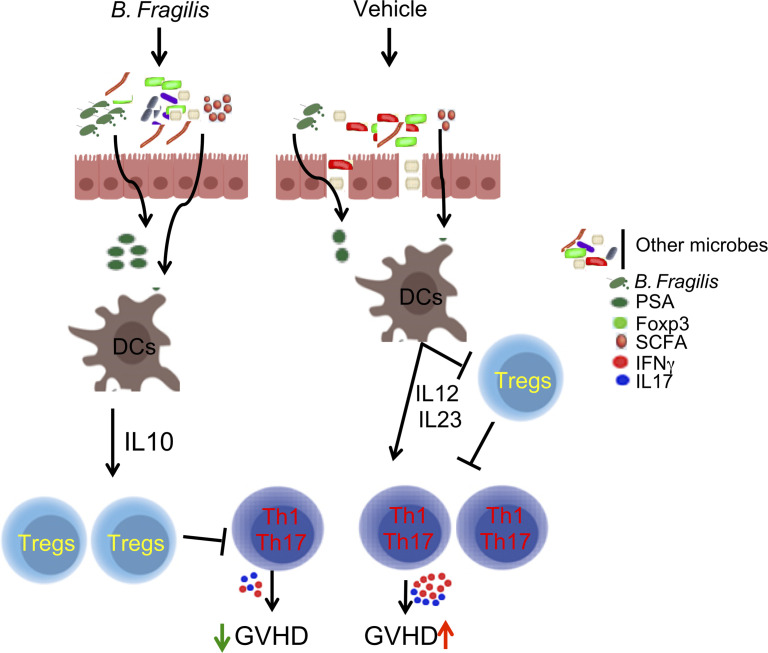

## Introduction

Graft-versus-host disease (GVHD) is a proinflammatory syndrome initiated by donor T cells and a major complication following allogeneic hematopoietic cell transplantation (allo-HCT) (1, 2). GVHD develops in 2 forms: acute (aGVHD) and chronic (cGVHD). aGVHD is primarily induced by T cells and is commonly characterized by a type 1 T cell response, whereas cGVHD is induced by both T and B cells and has similar manifestations to autoimmune disorders. During the acute phase, GVHD typically targets a restricted set of organs, including the skin, lung, liver, and gastrointestinal (GI) tract. Among these tissue sites, the GI tract is of particular importance in GVHD pathogenesis as damage to the gut plays a critical role in the initiation and amplification of systemic GVHD (3, 4). The consequence is attributable to breakdown of the mucosal barrier, which leads to augmented systemic proinflammatory cytokine production resulting from interactions between bacterial products (e.g., endotoxin) and immune cells that are residing in the host GI tract (5). Clinically, the gut injury itself and subsequent infectious complications can be life-threatening to the patient.

Microbiota refers to communities of microorganisms including bacteria, fungi, and viruses that are found in various body sites, including intestine (6, 7). Resident microbiota provides signals that facilitate normal immune system development and influence immune responses. On the other hand, the immune system shapes the composition of the microbiota and its proximity to host tissues. Thus, disruption of these dynamic interactions and mutualistic balance can have profound consequences on human health (8). The relationship between gut microbiota and GVHD development was recognized in the 1970s and 1980s, when GVHD risk was found to be substantially reduced in germ-free mice or in patients housed in a protective environment (9). However, rigorous examination of this relationship was only possible with recent advances in high-throughput sequencing technologies. Loss of bacterial diversity has been shown to be closely associated with GVHD severity in preclinical as well as clinical studies (6). More specifically, *Enterococcus spp*. potentially contributes to increased inflammation and reduced gut integrity, which was associated with more severe GVHD. In contrast, Clostridiales play an important role in antiinflammatory homeostasis, possibly due to upregulation of regulatory T cells (Tregs) through production of short chain fatty acids (SCFAs) (10–12). In fact, administration of butyrate enhanced the recovery of intestinal epithelium from damage after allo-HCT, and reintroduction of a mixture of 17 strains of human Clostridiales prolonged the survival of mice with GVHD (13).

Recently, increased attention has been paid to the use of prebiotics and probiotics to support the development and sustainability of a healthier microbiota, which potentially improves clinical outcomes and decreases rates of GVHD and posttransplant infection (14). Initial experiments and clinical trials have shown that fecal microbial transplantation (FMT) could be employed to preserve or restore the gut microbiota (15). However, it remains a daunting challenge to optimize FMT donors and to screen stool appropriately before FMT for immune-compromised patients. Thus, identifying and applying one or more live microorganisms that are safe and effective in improving the health of HCT patients are highly meritorious. In this study, we evaluated the impact of *Bacteroides fragilis*, a commensal bacterium that colonizes the mammalian GI tract, in the development of GVHD in murine models. We found that the abundance of *Bacteroides* in gut microbiota was negatively associated with severity of GVHD. Administration of *B*. *fragilis* through oral gavage increased gut microbial diversity and beneficial commensal bacteria including *Clostridium*, *Barnesiella*, *Bacteroides*, and *Lactobacillus* and decreased proinflammatory microbes including segmented filamentous bacteria and *Enterococcus*, thus creating a conducive diverse microbial environment that in turn significantly ameliorated the development of both aGVHD and cGVHD. These findings provide a strong rationale and potential means to prevent GVHD while preserving the graft-versus-leukemia (GVL) effect in the clinic using *B*. *fragilis* as a probiotic.

## Results

### FMT reduces aGVHD.

Loss of bacterial diversity is closely associated with GVHD severity in preclinical as well as clinical studies (6). As such restoration of host gut microbiota has been shown to alleviate GVHD in mice and patients after allo-HCT. An established approach to restore host microbiota is through FMT (6, 7). To test how FMT affects aGVHD development, we established a murine GVHD model in which recipient mice were treated with busulfan plus cyclophosphamide without total body irradiation (TBI), a treatment that mimics a conditioning regimen used in the clinic. We used naive C57BL/6 (B6) mice as a donor source of the FMT and compared the effects of FMT from the mice produced by Taconic Biosciences (Germantown, New York, USA) versus The Jackson Laboratory (Bar Harbor, Maine, USA), given the microbiota of these 2 sources has been defined previously (16, 17). Here we confirmed that B6 mice from Taconic and Jackson have distinct gut bacteria composition and diversity that are also reflected using principal coordinates analysis (PCoA) as shown in Supplemental Figure 1; supplemental material available online with this article; https://doi.org/10.1172/jci.insight.136841DS1 Furthermore, Taconic mice have increased abundance of gut-beneficial microbes including *Barnesiella* and *Lactobacillus*. Using an MHC-mismatched B6 BALB/c bone marrow transplantation (BMT) model, we found that the FMT from Taconic mice significantly alleviated GVHD severity as reflected by reduced weight loss, clinical scores, and mortality. In contrast the FMT from Jackson mice failed to protect recipients from GVHD (Supplemental Figure 2, A and B).

**Figure 1:**
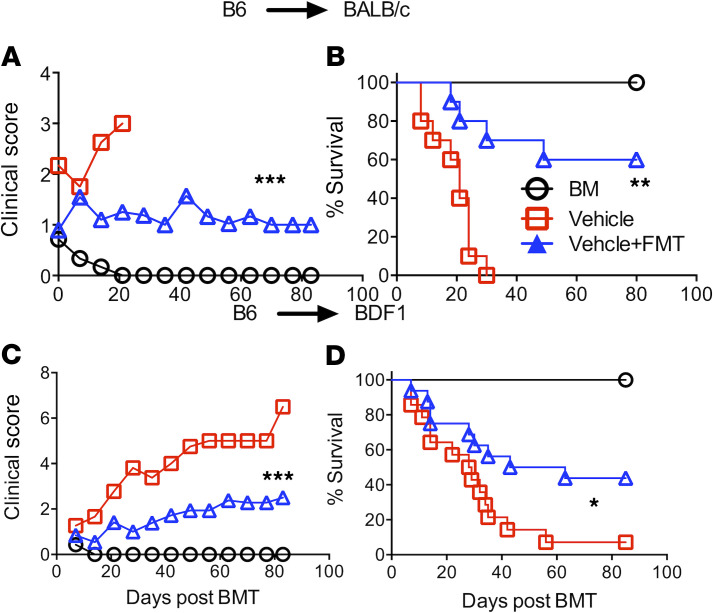
FMT from Taconic donor mice reduces GVHD. BALB/c or BD2F1 mice were treated with busulfan at 20 mg/kg and cyclophosphamide at 120 mg/kg daily for 6 days and then injected with 15 × 10^6^/mouse T cell–depleted BM (TCD-BM) alone or plus 15 × 10^6^ total splenocytes for BALB/c and 30 × 10^6^/mouse splenocytes for BD2F1 from normal B6 mice. The bedding of recipient cages was replaced with bedding without (control) or with fecal pellets from Taconic B6 mice. The FMT procedure was done thrice a week starting at 2 weeks prior to BMT and then weekly for a month post-BMT. Recipients were monitored for clinical score (**A** and **C**) and survival (**B** and **D**) (*n* = 9–15 per group). Data shown as mean ± SEM were pooled from 2 replicate experiments. Statistical tests: (**A** and **C**) 1-way ANOVA, (**B** and **D**) log-rank (Mantel-Cox). *P < 0.05, **P < 0.01, ***P < 0.001.

Given FMT only from Taconic B6 mice provided a beneficial effect on GVHD, we thereafter used Taconic mice as a donor source for FMT experiments. We substantiated the observation that the FMT from Taconic donor mice protected recipients from aGVHD in B6 BALB/c BMT model (Figure 1, A and B). Similar results were observed in a haploidentical B6 (B6 × DBA2)F1 (BDF1) BMT model (Figure 1, C and D). These results are in line with published observations that Taconic and Jackson mice have distinct microbiota, which leads to different host immune responses and impacts in cancer immunotherapy (16, 17).

### FMT affects T cell responses to alloantigens.

**Figure 2:**
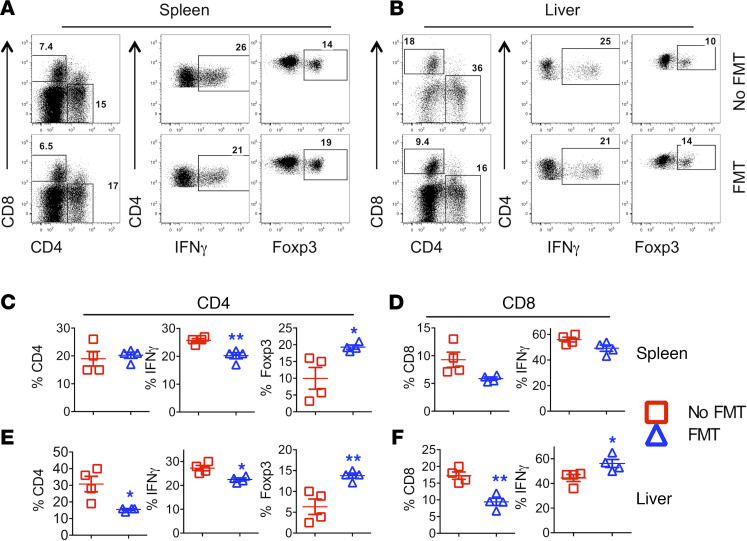
FMT effects on T cell activation and differentiation. B6 BALB/c BMT was initiated and FMT was administered as described in Figure 1. Three weeks after BMT, spleens and livers were collected from the recipients, and mononuclear cells were isolated and subjected to cell counting and FACS staining for surface H2K^b^ (donor MHC), CD4, CD8, and intracellular IFN-γ and Foxp3. CD4 and CD8 expression was shown on gated live H2K^b+^ donor cells (**A** and **B**). IFN-γ and Foxp3 (**A** and **B**) expression were shown on gated live H2K^b+^CD4^+^ donor cells. Data represent 1 of 3–5 mice in each group of recipients. The percentage of IFN-γ^+^ or Foxp3^+^ donor (H2K^b+^Ly5.1^–^) CD4^+^ (**C** and **E**), CD8^+^, and IFN-γ^+^ cells (**D** and **F**) in recipient spleen and liver are shown, respectively. Data shown as mean ± SEM are from 1 of 2 representative experiments. Significance was determined by Student’s *t* test. Asterisks indicate statistical significance *P < 0.05, **P < 0.01.

To understand the underlying mechanisms by which FMT reduces GVHD severity, we measured the activation, expansion, and differentiation of donor T cells in recipients’ spleen and liver 3 weeks post-BMT. The frequency and number of donor CD4^+^ and CD8^+^ T cells were similar in recipient spleens regardless of treatment (Figure 2, A, C, and D; and Supplemental Figure 3, A and B). However, administration of FMT significantly reduced the frequency and the number of CD4^+^ and CD8^+^ donor T cells in the liver of FMT recipients (Figure 2, B, E, and F; and Supplemental Figure 3, C and D, respectively), suggesting reduced migration of donor T cells to target organs. Donor CD4^+^ T cells in the liver also produced less IFN-γ after treatment (Figure 2, B, E, and F; and Supplemental Figure 3, C and D). In addition, administration of FMT significantly enhanced the frequency of Foxp3^+^ Tregs derived from donor CD4^+^ T cells in recipient spleen and liver (Figure 2, A–C and E). Taken together, administration of FMT ameliorated GVHD by reducing donor T cell expansion and Th1 differentiation in GVHD target organs while enhancing Treg generation or expansion.

### FMT influences recipient microbiota after allogenic BMT.

To address how FMT affects the recipient microbiota, we measured intestinal bacteria profiles in the tissue of recipients with or without FMT and found that *B*. *fragilis* was sharply increased among FMT recipients (Supplemental Figure 4, A and B) *B*. *fragilis* is a commensal bacterium that colonizes the mammalian GI tract and influences host immunity (18). These results suggested that the increase of *B*. *fragilis* in FMT recipients may contribute to GVHD reduction.

### B. fragilis ameliorates GVHD after allogeneic BMT.

**Figure 3:**
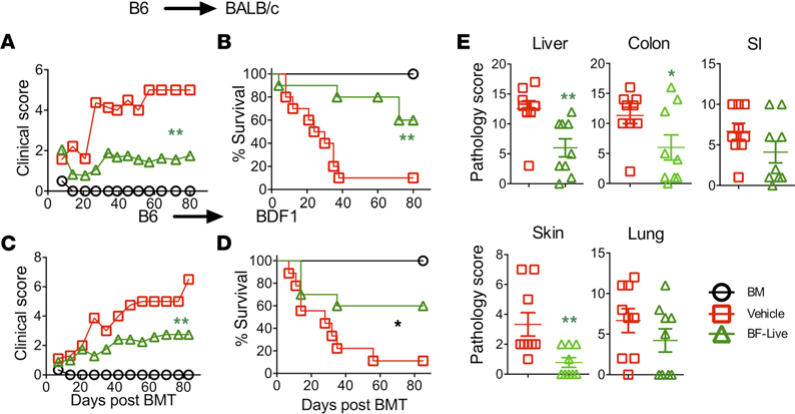
*B. fragilis* ameliorates GVHD. BALB/c (**A** and **B**) or BD2F1 (**C** and **D**) mice were preconditioned and BMT performed as described in Figure 1. The recipients were administered thrice a week through oral gavage with approximately 10^9^ live *B*. *fragilis* CFU (WT *B*. *fragilis*) from 2 weeks before BMT and then weekly until up to a month after BMT. Recipients were monitored for clinical score (**A**–**C**) and survival (**B**–**D**) until 80 days post-BMT (*n* = 10 per group). Data shown were pooled from 2 replicate experiments. In a similar setting, liver, lung, SI, colon, and skin were collected from the recipients 3 weeks post-BMT. Pathological score means ± SD of GVHD target organs are depicted (**E**). Data shown as mean ± SEM were pooled from 3 replicate experiments. Asterisks indicate statistical significance *P < 0.05, **P < 0.01.

Administration of *B*. *fragilis* has been shown to reduce the severity of autoimmune conditions such as colitis and type 1 diabetes (19, 20). We therefore asked whether *B*. *fragilis* could similarly affect GVHD development. Oral administration of live *B*. *fragilis* ameliorated GVHD in BALB/c recipients as reflected by lower clinical scores and a higher survival rate (Figure 3, A and B). Similar results were also observed in a haploidentical BMT model (Figure 3, C and D). We next asked if the colonization of *B*. *fragilis* is required for its impact on GVHD development. Interestingly administration of either live or heat-killed *B*. *fragilis* significantly ameliorated GVHD in BALB/c recipients as reflected by lower clinical scores and a higher rate of survival (Supplemental Figure 5, A and B). Clinical manifestations confirmed that recipients of *B*. *fragilis* had significantly less pathological damage in recipient liver, colon, and skin but not in small intestine (SI) and lung (Figure 3E). These data suggest that colonization may not be required for the *B*. *fragilis* protective effect on GVHD development.

### B. fragilis affects T cell responses to alloantigens.

**Figure 4:**
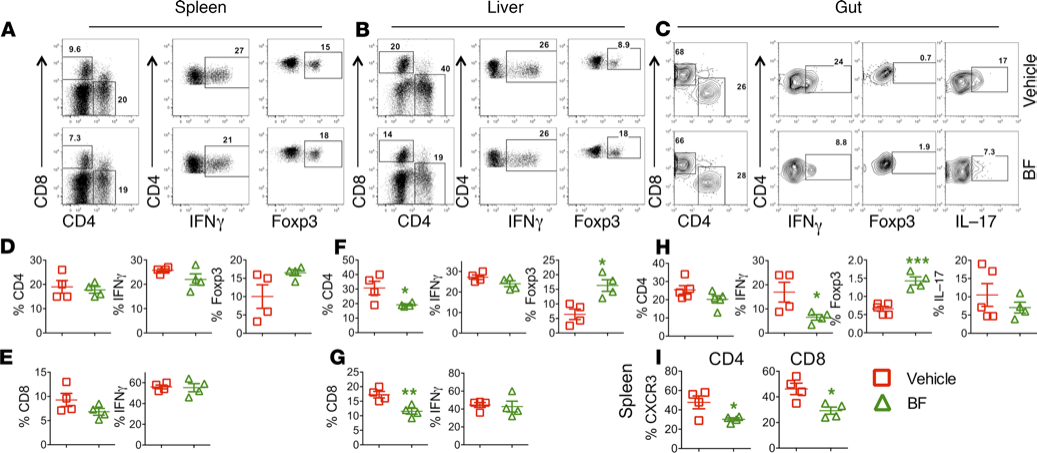
*B. fragilis* affects T cell activation and differentiation. B6 BALB/c BMT was initiated and *B*. *fragilis* was administered as described in Figure 3. Three weeks after BMT, spleen, liver, and gut (small and large intestine together) were collected from the recipients, and mononuclear cells were isolated and subjected to cell counting and FACS staining for surface H2K^b^ (donor MHC), CD4, CD8, and intracellular IFN-γ and Foxp3. CD4 and CD8 expression were shown on gated live H2K^b+^ donor cells (**A**–**C**). IFN-γ, Foxp3, and IL-17 expression were shown on gated live H2K^b+^CD4^+^ donor cells and IFN-γ on gated live H2K^b+^CD8^+^ donor cells. The percentage of IFN-γ^+^, Foxp3^+^ from (H2K^b+^Ly5.1^–^) CD4^+^ (**D** and **F**) and IFN-γ^+^, Foxp3^+^, and IL-17^+^ from (H2K^b+^Ly5.1^–^) CD4^+^ T cells shown in recipient spleen, liver, and (**H**) gut, respectively. IFN-γ from CD8^+^ T cells shown in recipient spleen and liver, respectively (**E** and **G**). Percentage CXCR3^+^ cells is summarized on donor-derived CD4^+^ and CD8^+^ cells in recipient spleen (**I**). Data shown as mean ± SEM are from 1 of 2 representative experiments. Significance was determined by Student’s *t* test. Asterisks indicate statistical significance *P < 0.05, **P < 0.01, ***P < 0.001.

Development of GVHD requires donor T cell activation and expansion in lymphoid organs and subsequent migration into target organs (21). Hence, we asked whether *B*. *fragilis* affects T cell infiltration and expansion into target organs. Three weeks after BMT, we examined the presence of donor T cells in recipients’ spleen (secondary lymphoid organ), liver, and gut (gut includes small and large intestine) (GVHD target organs). Using the gating strategy shown in Supplemental Figure 6, we found that the frequency and the number of donor CD4^+^ and CD8^+^ T cells were similar in recipient spleens regardless of treatment (Figure 4, A, D, and E; and Supplemental Figure 7, A and B, respectively). Administration of *B*. *fragilis* significantly reduced the frequency and number of CD4^+^ and CD8^+^ donor T cells in the liver (Figure 4, B, F, and G; and Supplemental Figure 7, C and D, respectively), suggesting the treatment reduced expansion or migration of donor T cells in target organs. Donor CD4^+^ T cells that migrated to gut (both small and large intestines) had reduced frequency of IFN-γ production after treatment with *B*. *fragilis* (Figure 4, C and H), *and* both liver and gut had significantly reduced IFN-γ CD4^+^ T cell numbers (Supplemental Figure 7, C and E). Of note, IL-17 frequency was also decreased in the gut, although the difference was not statistically significant (Figure 4, C and H). In addition, administration of *B*. *fragilis* significantly enhanced the frequency of Foxp3^+^ Tregs derived from donor CD4^+^ T cells in spleen, liver, and guts (Figure 4, A–C, F, and H). We further hypothesized that *B*. *fragilis* may reduce T cell migration to GVHD target organs. Indeed, T cells from *B*. *fragilis–*administered recipients had significantly reduced expression of CXCR3 compared with vehicle counterparts in the spleen (Figure 4I). Hence, we interpret that *B*. *fragilis* reduces T cell activation and proliferation, and thus these T cells have compromised migratory potential to target organs.

### Impact of FMT or B. fragilis on recipient microbiota after allogenic BMT.

**Figure 5:**
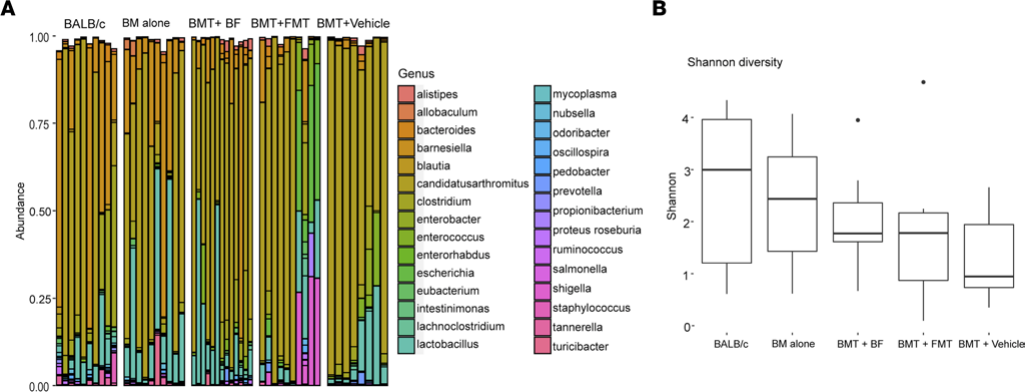
Impact of FMT and *B. fragilis* on recipient microbiota after allogenic BMT. B6 BALB/c was initiated and FMT and *B*. *fragilis* were administered as described in Figure 1 and Figure 3, respectively. Thirty days after BMT, ileums were collected from the recipients and extracted for total DNA, which was used for 16S rRNA sequencing. (**A**) Bacterial composition of abundantly expressed (>1% abundance in at least 1 sample) genus-level taxa is depicted, and nonparametric 1-way Kruskal-Wallis ANOVA identifies 12 genera to be statistically different after false discovery correction (*q* < 0.1). (**B**) Diversity among the groups is shown by Shannon diversity index, which demonstrates a statistically significant linear trend (*P* < 0.007).

To address whether FMT or *B*. *fragilis* affects the recipient gut microbiota, we collected ileums from 4 groups of recipients that were transplanted with BM alone (BM alone), BM plus splenocytes and treated with vehicle (BMT + vehicle), BM plus splenocytes and treated with FMT (BMT + FMT), or BM plus splenocytes and treated with *B*. *fragilis* (BMT + BF) 30 days after BMT. A group of unmanipulated BALB/c mice were also used as a baseline control. DNA was extracted from ileum and used for 16S rRNA sequencing. The microbiota in normal BALB/c mice showed the highest bacterial diversity as expected and similarly in the BALB/c recipients of BM alone group (Figure 5, A and B). In contrast, the microbiota in BALB/c recipients of BMT with vehicle showed the least bacterial diversity. FMT and more BF administration was able to increase bacterial diversity (Figure 5, A and B). Among 5 groups, the trend of bacterial diversity according to the Shannon alpha diversity measure was statistically different (trend test, *P* < 0.007), which was inversely correlated with GVHD severity. Furthermore, beneficial commensal bacteria including *Barnesiella*, *Lactobacillus*, *Clostridium*, and *Bacteroides* exhibited an increased trend in the recipients treated with FMT or *B*. *fragilis* compared with vehicle. On the other hand, some proinflammatory microbes, including segmented filamentous bacteria and *Enterococcus*, showed a downward trend in these groups compared with the vehicle group. However, the differences were not significant after adjustment for false discovery rate (*q* > 0.1). These results suggest that *B*. *fragilis* or FMT may create a conducive microbial environment that in turn influences the outcome of GVHD development.

### Polysaccharide A is required for B.fragilis to protect GVHD development.

*B*. *fragilis* reduces autoimmune disease via polysaccharide A (PSA), which can regulate immune responses (18). To test whether PSA is required for *B*. *fragilis* to ameliorate GVHD, we evaluated the effect of PSA-deficient *B*. *fragilis*. While administration of live or heat-killed *B*. *fragilis* significantly reduced GVHD, the PSA-deficient *B*. *fragilis* failed to protect recipients from GVHD (Supplemental Figure 8, A and B). These data suggest that PSA is an important component in order for *B*. *fragilis* to ameliorate GVHD.

### B. fragilis–derived metabolites affect gut integrity during GVHD development.

**Figure 6:**
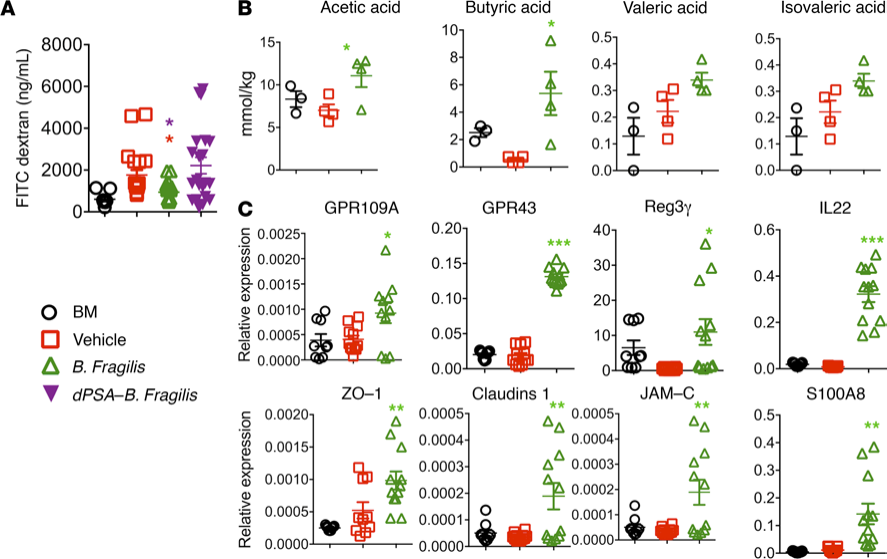
*B. fragilis* increases SCFAs in the recipient intestine after BMT. B6 BALB/c BMT was initiated and *B*. *fragilis* was administered as described in Figure 3. BALB/c recipients were administered FITC-dextran by oral gavage and deprived of food and water for 4 hours on day 7 post-BMT. Peripheral blood was collected from the recipients and concentrations of FITC-dextran were measured (**A**). Data shown are from 2 representative experiments with each mouse sample in triplicates of 4–8 mice from each group. Three weeks after BMT, recipient ileums were collected and snap-frozen in dry ice. SCFAs were measured in intestinal tissues. The expression of different SCFAs are shown (**B**). The data shown here are from 1 of the 2 representative experiments with 3–4 mice from each group. The quantitative expression of different genes is shown (**C**). Data from 1 of the 2 representative experiments with each mouse sample in triplicates of 3–4 mice from each group. Significance is determined by 1-way ANOVA (using multiple comparison test). Asterisks indicate statistical significance *P < 0.05, **P < 0.01, **P < 0.001.

The microbiota and its products have been shown to control T cell–dependent inflammation through several mechanisms, including maintaining gut integrity and the conversion of precursors provided by the diet into immune-regulatory metabolites (22–24). Based on this, we first examined how *B*. *fragilis* affects intestinal barrier function after allo-BMT using an FITC-dextran assay. Dextran is a nonmetabolized carbohydrate that can enter the bloodstream if the epithelial barrier has been compromised. Administration of *B*. *fragilis* significantly reduced the serum levels of FITC-dextran (Figure 6A) versus vehicle controls. In contrast, the impact of PSA-deficient *B*. *fragilis* was minimal (Figure 6A), suggesting that *B*. *fragilis* maintained intestinal barrier function and ameliorated GVHD through a PSA-dependent manner. As SCFAs are a well-known group of metabolites that regulate intestinal function (25), we tested the impact of *B*. *fragilis* on SCFA production after allo-BMT. Among various SCFAs, including acetic, propionic, butyric, valeric, isovaleric, hexanoic, isobutyric, and heptanoic acids, we observed that administration of *B*. *fragilis* significantly increased the levels of acetic acid and butyric acid in recipient ileums 3 weeks after allo-BMT (Figure 6B). To test the effect of *B*. *fragilis* on gut epithelial function, we isolated RNA from the gut tissues and performed quantitative PCR. A significant increase in GPR109A, GPR43, Reg3γ, and IL-22 was observed in recipients treated with *B*. *fragilis* (Figure 6C). S100A8 as well as these 4 molecules have been known for their antibacterial potential via their ability to bind Zn^2+^. These molecules are known to be associated with the protection of intestinal epithelium during inflammatory conditions. Furthermore, we examined several gut tight junction–associated proteins and found a drastic increase in Zona Occludens1 (ZO-1), Claudins1, and Junctional adhesion molecule 3 (JAM-C) after *B*. *fragilis* administration (Figure 6C). These data suggest an important role of *B*. *fragilis* in regulating gut integrity likely through SCFA production, which in turn mitigates GVHD development.

### B. fragilis improves recipient survival after gut decontamination using broad-spectrum antibiotics.

**Figure 7:**
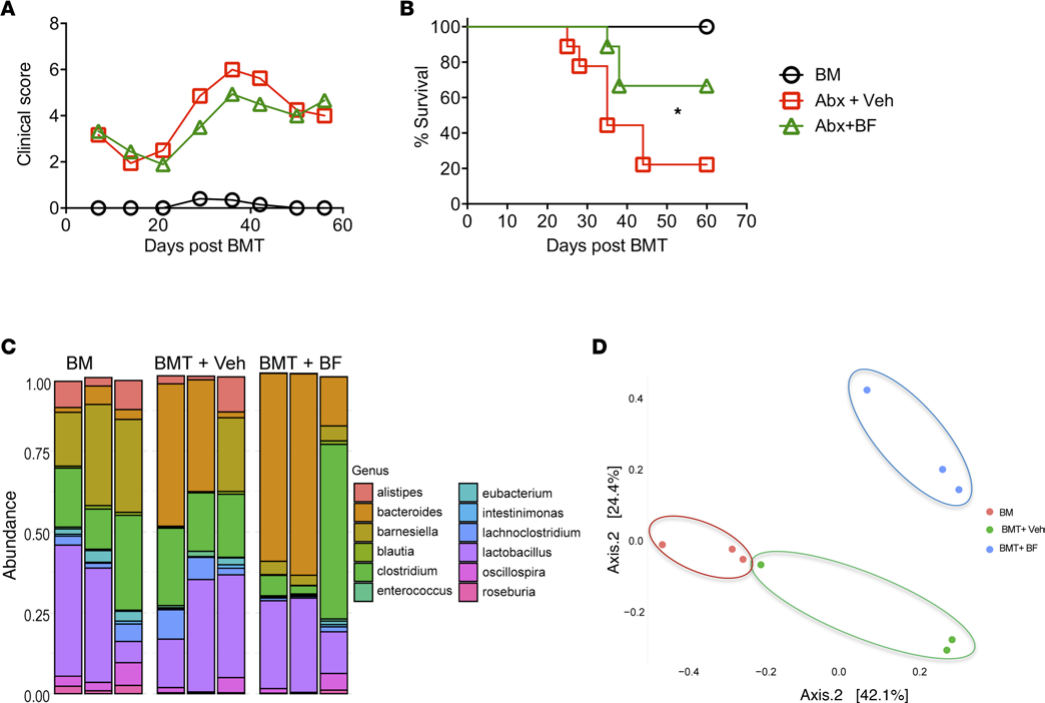
Reshaping commensal microbiota using *B. fragilis* reduces GVHD. Recipient BALB/c mice were treated with a cocktail of broad-spectrum antibiotics for 21 days followed by 2 days of rest. A group of these recipients were administered through oral gavage with either vehicle or live *B*. *fragilis* as described in Figure 1. Recipients were monitored for clinical score (**A**) and survival (**B**) until 60 days post-BMT (*n* = 10 per group). Data shown are from 2 combined experiments. (**C**) Fecal pellets were collected from the recipients on day 30 after BMT and extracted for total DNA, which was used for 16S rRNA sequencing. Data shown are from 1 representative experiment. One-way ANOVA analysis of center log ratio–transformed abundances of major genera (present at >1% in at least 1 sample) is shown (*P < 0.05). (**D**) PCoA of the Bray-Curtis distances indicates visual and statistically significant separation according to Wd* test (*P* < 0.007).

We next asked whether *B*. *fragilis* requires other commensal bacteria to ameliorate GVHD. Broad-spectrum antibiotics are frequently used for gut decontamination, which also leads to the changes in the composition and diversity of the intestinal microbiota (26–28). BALB/c mice were orally gavaged with a broad-spectrum antibiotic cocktail for 21 days. These mice were then used as BMT recipients for testing the effect of *B*. *fragilis* on GVHD development as described in Figure 3. We observed that administration of live *B*. *fragilis* still ameliorated GVHD in BALB/c recipients as reflected by significantly higher survival rate (Figure 7, A and B). Fecal pellets were collected on day 30 post-BMT and characterized for 16S rRNA sequencing. The frequency of *Bacteroides* was 26.8% in the recipients treated with vehicle but was increased to 45.1% in those treated with *B*. *fragilis* (Figure 7C). PCoA indicated that bacterial composition was significantly different among the 3 groups (*P* < 0.007, Figure 7D). That orally gavaged bacteria can reach to the intestine and colonize was also observed previously (29). These results support that *B*. *fragilis* can colonize and ameliorate GVHD severity after allogeneic BMT in recipients with gut decontamination.

### Administration of B. fragilis preserves the GVL activity.

**Figure 8:**
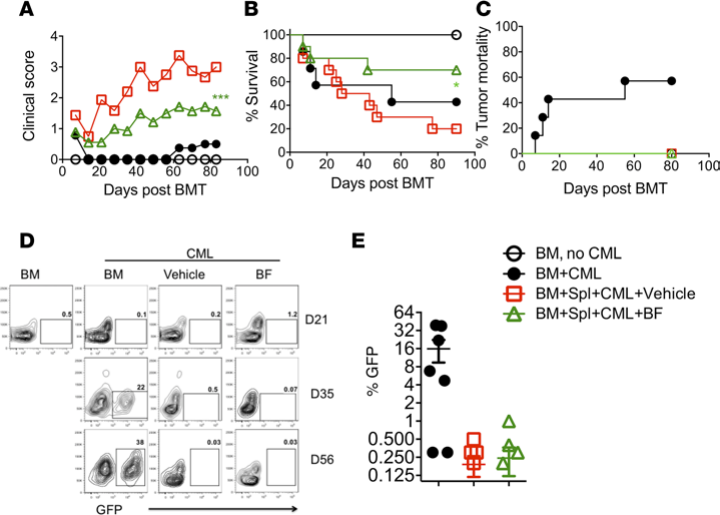
*B. fragilis* effects on GVL response. B6→BALB/c BMT was initiated and recipients were treated as shown in Figure 3 with or without BC-CML cells at a dose of 1 × 10^6^ BC-CML cells per mouse. Recipients were monitored for clinical score (**A**), survival (**B**), and (**C**) tumor mortality until 80 days post-BMT (*n* = 10 per group). Peripheral blood from recipients was collected periodically starting 21 days after transplant and analyzed via flow cytometry for expression of GFP^+^ BC-CML cells. Percentage of GFP^+^ cells is shown in recipient blood (**D** and **E**). Data shown as mean ± SEM were pooled from 2 replicate experiments. Significance is determined by 1-way ANOVA (using multiple comparison test). Asterisks indicate statistical significance *P < 0.05, ***P < 0.001.

We next asked whether *B*. *fragilis* administration attenuated GVHD while maintaining GVL activity. To test this, we used a B6→BALB/c BMT model with blast crisis chronic myelogenous leukemia (BC-CML) model. As expected, *B*. *fragilis* reduced GVHD severity reflected by clinical score and survival (Figure 8, A–C). Among the recipients that received BM alone with BC-CML, 60% of mice had tumor relapse and died from the tumor as reflected by GFP^+^ cells in peripheral blood and tumor mortality (Figure 8, C–E). The recipients of donor BM plus splenocytes and treated with vehicle or *B*. *fragilis* did not display any signs of tumor growth in the peripheral blood at any time point tested (Figure 8, C–E). Taken together, these data suggest administration of *B*. *fragilis* preserves the GVL activity while ameliorating GVHD.

### B. fragilis is effective in preventing cGVHD after allo-BMT.

**Figure 9:**
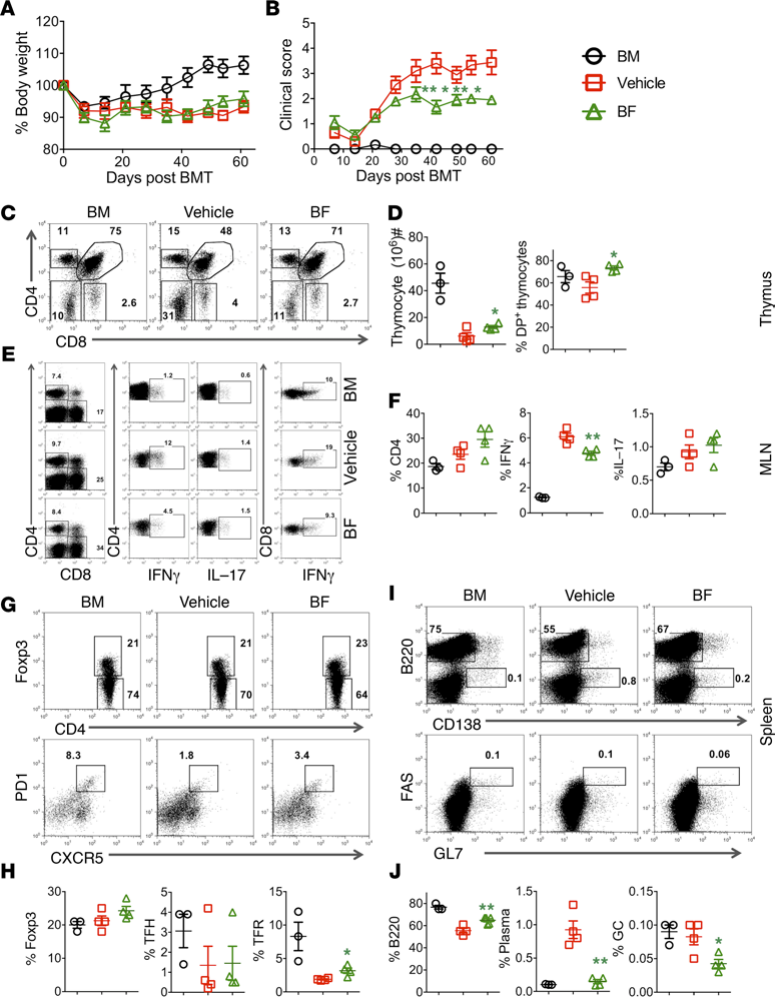
*B. fragilis* is effective in preventing cGVHD. BALB/c mice were lethally irradiated and transplanted with 5 × 10^6^/mouse TCD-BM (Ly5.1^+^) alone or plus purified splenocytes (Ly5.2^+^) (0.5 × 10^6^/mouse) from B6 mice. The recipients were administered live *B*. *fragilis* 3 times a week through oral gavage with approximately 10^9^ live *B*. *fragilis* CFU for 1 week before BMT, then once a week until up to 1 month after BMT. Recipients were monitored for body weight (**A**) and clinical score (**B**) until 60 days post-BMT (*n* = 10 per group, 1-way ANOVA). Absolute numbers and phenotypes of recipient thymocytes were determined. The percentages of CD4^+^/CD8^+^ double-positive thymocytes (**C**) and total thymocytes (**D**) are shown. The percentages of IFN-γ^+^ and IL-17^+^ on donor CD4^+^ and IFN-γ^+^ on CD8^+^ cells are shown in recipient lymph nodes (MLNs) (**E**). Percentage IFN-γ^+^ and IL-17^+^ on gated donor CD4^+^ cells is shown in recipient MLNs (**F**). (**G**) Expression of Foxp3^+^ cells on gated donor CD4^+^ and follicular regulatory cells and (**H**) percentage of regulatory T cells, follicular regulatory T cells (Foxp3^+^CXCR5^+^PD-1^+^), and follicular helper T cells (Foxp3^–^CXCR5^+^PD-1^+^). (**I** and **J**) Splenic B cells were analyzed for B220^+^ and B220^−^CD138^+^, respectively. Data shown as mean ± SEM are from 1 of the 2 replicate experiments. Asterisks indicate statistical significance *P < 0.05, **P < 0.01.

cGVHD remains a major cause of mortality and morbidity after allo-HCT. cGVHD pathology is characterized by autoimmune-like, multiorgan fibrosis, exhibiting features like scleroderma and bronchiolitis obliterans. The pathogenesis of cGVHD involves impaired Treg generation, as well as aberrant follicular helper T cell differentiation, germinal center (GC) formation, and auto/allo-antibody production. Given cGVHD has a distinct pathophysiology from aGVHD, we evaluated the effect of *B*. *fragilis* on cGVHD development. Administration of *B*. *fragilis* prevented cGVHD as reflected by a significant reduction in clinical score (Figure 9, A and B).

In cGVHD, host thymus is also a GVHD target (30). We thus examined how *B*. *fragilis* affects thymocyte reconstitution. Consistent with severe cGVHD, recipients treated with vehicle had substantially reduced total thymocytes and double-positive cells compared with recipients of BM alone (Figure 9, C and D). Administration of *B*. *fragilis* protected the host thymus from damage as reflected by increased total thymocytes and double-positive cells (Figure 9, C and D). We next determined the effect of *B*. *fragilis* on donor T cell differentiation by measuring IFN-γ and IL-17 on day 60 after allo-BMT and found that donor CD4^+^ T cells produced significantly less IFN-γ, but not IL-17, in the mesenteric lymph nodes (MLNs), although no difference was observed in the spleen (data not shown) (Figure 9, E and F). In addition we observed a significant increase in follicular regulatory T cells with no significant difference on follicular T helper cells as shown (Figure 9, G and H). However, the CD8 cytokine levels were similar in both compartments. Given donor B cells are pathogenic yet also target cells in cGVHD, we evaluated the effect of *B*. *fragilis* on donor B cell reconstitution and differentiation. Administration of *B*. *fragilis* improved donor B cell reconstitution versus vehicle controls to a level comparable to that of BM alone recipients (Figure 9, I and J). Plasma cells (CD138^+^B220^lo^) and GC B cells (B220^+^Fas^+^GL7^+^) are pathogenic in cGVHD. We found that administration of *B*. *fragilis* substantially reduced GC B cells and plasma cells (Figure 9, I and J) in recipient spleens. These results suggest that *B*. *fragilis* administration alleviates cGVHD severity through reducing Th1 responses and pathogenic B cell differentiation.

## Discussion

Advances in microbial analysis have provided new insights into the complex interactions between the host and gut microbiota. The community structure of the microbiota is altered after allo-BMT (31) and is also associated with various disorders and their pathogenesis. Furthermore, increasing evidence indicates that the gut microbiota is closely associated with GVHD and transplant outcomes, suggesting that its manipulation could be a new treatment strategy to regulate GVHD development (13). In the current report, we evaluate the effect of FMT on the development of aGVHD and observed that increased *Bacteroides* in recipient gut microbiota was associated with reduced GVHD after FMT. Furthermore, we demonstrate that administration of a *B*. *fragilis* isolate enhanced gut diversity and beneficial commensal bacteria while decreasing proinflammatory bacteria, which in turn creates a conducive diverse microbial environment leading to improved gut integrity and reduced GVHD development largely in a PSA-dependent manner. Administration of *B*. *fragilis* also increased SCFAs, especially butyric acid and acetic acid in gut tissues, and enhanced intestinal tight junction integrity. Moreover, administration of *B*. *fragilis* was able to prevent cGVHD and preserve the GVL activity. Our finding provides evidence that a single strain of bacteria, namely *B*. *fragilis*, is capable of dampening recipient gut injury and thus ameliorating GVHD development.

Commensal bacteria and probiotics have been shown to exert their antiinflammatory effect through production of IL-10, and the Th2-associated cytokines, such as IL-25 and IL-33, as well as via induction of regulatory cells (32, 33). There are several putative mechanisms by which microbiota therapy could affect inflammation distal from the gut, including production of regulatory cytokines, expansion/generation of Tregs or through suppressive DCs. There may also be an expansion of regulatory cells that traffic to the site of inflammation and affect gut permeability (34). Under homeostatic conditions, the gut environment is conducive to induction of regulatory cells, which is likely disturbed in an inflammatory atmosphere such as seen in GVHD. Administration of *B*. *fragilis* was observed previously to be protective against inflammatory colitis (35–37). Our current study demonstrates that *B*. *fragilis* was capable of preventing GVHD. We observed that *B*. *fragilis* reduced activation of pathogenic T cells and their impeded migration to target organs, such as the liver and gut, while increasing generation or expansion of Tregs (Figure 4). These data suggest that the presence of *B*. *fragilis* improves gut health and in turn lessens GI GVHD development.

The intestinal injury caused by conditioning regimens results in increased intestinal permeability that facilitates translocation of gut bacteria over the intestinal barrier. As a result, immune stimulation is augmented in response to a range of pathogens, which reinforces inflammatory cytokine response and provides an ideal setting for allogeneic T cell activation and priming (28, 38, 39). Recent reports indicate that the modulation of gut microbiota may represent a potential therapeutic intervention to alleviate and even prevent GVHD in allo-HCT patients (38). The gut microbiota can be manipulated by the use of probiotics to repopulate the gut with commensals (38). Several studies have reported the use of commensal bacteria and probiotics to promote intestinal barrier integrity in vivo (40–43), competitive exclusion to colonization from many other pathobionts, and production of SCFAs as well as vitamins (44, 45). We observed many of the aforementioned features after *B*. *fragilis* administration, which decreased gut inflammation, protected intestinal barrier function, and reduced gut permeability.

PSA-deficient *B*. *fragilis* essentially lost its protective activity (Supplemental Figure 8), whereas heat-killed *B*. *fragilis* was still effective in ameliorating GVHD (Supplemental Figure 5). We reason that *B*. *fragilis* exerts its protective activity largely in a PSA-dependent manner. Our observation is consistent with published reports showing that PSA is a critical component for the beneficial effect of *B*. *fragilis* in other diseases (46, 47). Heat-insensitive PSA is known to modulate host microbiota and immunity by reducing the inflammatory response and provides protection from inflammatory cell infiltration into gut, thus reducing gut damage and preserving gut integrity (35). The notion that *B*. *fragilis* exerts its protective activity largely through PSA-dependent mechanism is supported by our observation that *B*. *fragilis* was protective on GVHD in the recipients receiving broad-spectrum antibiotics (Figure 7), although efficacy was lower when compared with recipients not receiving selective digestive tract decontamination (Figure 3). These results suggest that other microbiota in recipient gut may contribute to *B*. *fragilis*–mediated beneficial effect besides PSA.

It has been shown that GVHD suppresses intestinal REG3γ expression, and the absence of REG3γ in BMT recipients intensified GVHD (48). The principal inducer of REG3γ in both Paneth cells and other enterocytes is IL-22, which is produced by innate lymphoid cells. Administration of IL-22 induced REG3γ and protected intestinal stem cells (ISCs), preventing a breach in the gut epithelial barrier and reducing gut GVHD (49). We observed that IL-22 and REG3γ gene expression was significantly increased in gut tissues of the recipients treated with *B*. *fragilis*. Therefore, we interpret that *B*. *fragilis* increased IL-22 production and in turn protected ISCs and epithelial barrier function, which is supported by elevated tight-junction regulated proteins including ZO-1, Claudins1, and JAM-C (Figure 5). These studies further strengthen the premise of using beneficial microbes not only to restore gut homeostasis by modulating microbiota diversity but also to preserve gut integrity.

The interplay between gut commensal bacteria and the host provides beneficial effects on host metabolism as well as immune regulation (50). Microbiota-derived metabolites heavily influence intestinal homeostasis and affect both nonimmune and immune cells. For instance, some isolates of Clostridia, known to produce SCFAs such as butyrate, increase frequencies of Tregs in the intestinal tract (10, 12), which in turn suppress GVHD (51–53). Similarly, we observed that administration of *B*. *fragilis* increased butyrate levels and its cognate receptor’s (GRPR43) expression. Among SCFAs, butyrate plays an important role in maintaining intestinal barriers (13, 54). Butyrate serves as an energy source for intestinal epithelial cells and induces regulatory immune responses locally and systemically (55–57). Consistently, increased butyrate and butyrate receptor were associated with decreased gut-inflammatory cytokines, especially IFN-γ, and increased frequency of Tregs. Therapies capable of inducing a shift toward an antiinflammatory atmosphere may be of value to control the incidence and severity of GVHD.

Outside of the effect on T cells, administration of *B*. *fragilis* also restrained B cell activation and maturation into plasma and GC B cells. Although it is not clear whether the impact on B cells was a direct effect of *B*. *fragilis* or via T cells, we interpret that *B*. *fragilis* administration resulted in amelioration of cGVHD (Figure 7). A recently published report further supports this by showing that gut-resident B cells bound PSA and B cells were crucial for induction of Tregs secreting IL-10 that are critical for restraining pathogenic innate inflammatory responses (46). Notably, administration of *B*. *fragilis* preserved the GVL effect while preventing GVHD (Figure 6). Underlying mechanisms may be several fold, including (a) administration of *B*. *fragilis* protects gut injury and thus reduces gut GVHD without inducing systemic immunosuppression, hence enabling maintenance of the GVL effect; (b) *B*. *fragilis* significantly increased frequency of induced Tregs, which in turn may be prone to suppressing GVHD over GVL responses by largely sparing the perforin killing pathway (58); and (c) *B*. *fragilis* significantly reduced migration of donor T cells into target organs (e.g., liver), potentially through reduced CXCR3, whereas it did not affect T cell expansion or proinflammatory cytokine production, especially IFN-γ, in the lymphoid organs (Figure 4 and Supplemental Figure 7). Thus, activated T cells in the refined tissues could still exert their GVL response without causing severe injury in parenchymal tissues.

In conclusion, we demonstrate that treatment with *B*. *fragilis* reduces acute and chronic GVHD. The reduced GVHD was associated with several changes in T cell and B cell alloresponses induced by *B*. *fragilis* via a PSA-dependent manner. *B*. *fragilis* modulates these responses likely by stabilizing the gut environment through SCFA-mediated mechanisms. As such, recipient gut integrity is maintained and can be partially attributed to intestinal crypt regeneration via IL-22 and GRP43, ultimately shifting the immune response toward reduced inflammation, resulting in GVHD protection. This study provides a strong rationale and means for the use of a single bacterial strain (such as *B*. *fragilis*) as a safe and effective intervention that would be of benefit to allo-HCT patients in the clinic.

## Methods

### Mice.

C57BL/6 (B6; H-2^b^, CD45.2), B6.Ly5.1 (CD45.1), BD2F1 (H-2^b/d^), and BALB/c (H-2^d^) mice were purchased from the National Cancer Institute/NIH (Frederick, Maryland, USA), The Jackson Laboratory (Bar Harbor, Maine, USA), or Taconic Biosciences (Germantown, New York, USA). All animals were housed in the Medical University of South Carolina (MUSC). Experiments were carried out under protocols approved by the Institutional Animal Care and Use Committee of MUSC.

### Microbial growth conditions.

*B*. *fragilis* was purchased from ATCC (catalog 25285). Isogenic *B*. *fragilis* deficient for PSA (ΔPSA-BF) was provided by Laurie E. Comstock (Harvard Medical School, Boston, Massachusetts, USA). Both strains were grown as per the ATCC propagation protocol. Briefly, *B*. *fragilis* was cultured from a single colony in brain-heart infusion (BHI) medium under anaerobic conditions for up to 72 hours at 37°C, diluted 50-fold using complete medium, and grown for an additional 16 hours at 37°C. Bacterial cells were pelleted and washed with PBS twice by centrifugation at 20,000*g* for 20 minutes at 4°C, aliquoted, and frozen in 30% glycerol stock as live bacteria until use. Some of the frozen aliquots were heat inactivated by incubation at 65°C for 30 minutes. Heat-inactivated bacteria were tested for viability by plating and culturing on BHI agar plates for 72 hours at 37°C. Heat-inactivated bacteria were subjected to an additional quick wash in sterile distilled water to minimize salt content, and the pellet was air-dried and its dry weight determined, before suspending at 50 mg/mL (*w/v*) PBS, as stock suspension.

### BMT.

MHC-mismatched (B6→BALB/c) and haploidentical (B6→BD2F1) BMT models were used to establish a murine GVHD model in which recipient mice were treated with busulfan plus cyclophosphamide or with TBI. When chemotherapy was used as conditioning, mice were injected with busulfan (20 mg/kg/d) and cyclophosphamide (120 mg/kg/d) from day –7 to day –2 (6 days that include 4 full doses and last 2 half doses) daily administered intraperitoneally as reported previously (59, 60). The conditioning regimen was designed to be similar to a clinical setting. After BMT, recipient mice were monitored for weight loss and other clinical signs of GVHD twice per week. Clinical scores were tabulated based on 5 parameters, including weight loss, posture, activity, fur texture, and skin integrity, for aGVHD and 7 parameters for cGVHD, including weight loss, posture, activity, fur texture, skin integrity, diarrhea, and eyes swollen and difficult to open (61, 62). Individual mice were scored from 0 to 2 for each criterion and from 0 to 10 overall for aGVHD and 0 to 14 for cGVHD. Recipients at the premorbid stage were euthanized and counted toward lethality. Recipients at premoribund stage were euthanized and counted for lethality. Representative samples of GVHD target organs were excised from recipients 21 days after BMT and subjected to pathology scoring and other immunological analysis. In a different set of experiments, SPF BALB/c mice (from National Cancer Institute/NIH, Frederick, Maryland, USA) were treated with a cocktail of broad-spectrum antibiotics (4 mg of ampicillin, 4 mg of neomycin, 2 mg of vancomycin, and 2 mg of metronidazole) (MilliporeSigma), daily by oral gavage for 21 days to target host microbes. Two days later these mice were orally gavaged with either *B*. *fragilis* or the vehicle every alternate day for 1 week and then weekly for 30 days post-BMT. Animals were monitored for development of clinical score and survival.

### GVL response.

The BC-CML model was generated previously (63) and provided by Warren D. Shlomchik (University of Pittsburgh, Pittsburgh, Pennsylvania, USA). The MHC-mismatched (B6→BALB/c) BMT model was used to establish a murine GVHD model in which recipient mice were treated with busulfan plus cyclophosphamide or with TBI with or without BC-CML cells at a dose of 1 × 10^6^ BC-CML cells per mouse together with TCD-BM (15 × 10^6^ per mouse) with or without whole splenocytes (15 × 10^6^ per mouse). Peripheral blood from recipients was collected periodically starting 21 days after transplant and analyzed via flow cytometry for expression of GFP^+^ BC-CML cells.

### Flow cytometry.

Mononuclear cells were isolated from mouse recipient spleen or liver. LIVE/DEAD fixable yellow from Invitrogen, Thermo Fisher Scientific (catalog L34968), was used to distinguish live and dead cells. The isolated cells were stained for surface markers and intracellular cytokines using standard protocols. Stained cells were analyzed using FACSDiva software, LSR II (BD Biosciences), and FlowJo (Tree Star). The following antibodies were used for cell surface staining: anti-CD4-V450, -APC, and -PEcy7 (RM4-5, BD Biosciences); anti-CD8-PEcy5, -APCcy7, and -AF700 (53-6.7, BD Biosciences); anti-CD45.1-FITC and -APC (A20, BD Biosciences); anti-B220-FITC and -PE (RA3-682, eBioscience, Thermo Fisher Scientific); anti-CD44-APC and -PE (IM7, eBioscience, Thermo Fisher Scientific); anti-CD62L-PEcy5 and -FITC (MEL-14, eBioscience, Thermo Fisher Scientific); CD25-FITC (7D4) and -PEcy7 (PC 61.5, eBioscience, Thermo Fisher Scientific); and anti-H2K^b^ (AF6-88.5.5.3). Intracellular staining was carried out using anti–IFN-γ-PE or Per-cp 5.5 (XMG1.2, BD Biosciences), anti–IL-4-PE (11B11, BD Pharmingen), anti–IL-5-PE (TRFK5, BD Pharmingen), and anti-Foxp3-PE (FJK-16s, eBioscience, Thermo Fisher Scientific).

### Histologic analysis.

Representative samples of liver, SI, large intestine, lung, and skin were obtained from transplanted recipients 21 days posttransplant, fixed in 10% neutral-buffered formalin, and washed with 70% ethanol. Samples were then embedded in paraffin, cut into 5 μm thick sections, and stained with H&E. A semiquantitative scoring system was used to account for histologic changes consistent with GVHD in the colon, liver, and lung as described previously (61, 62). Data were presented as individual GVHD target organs. All slides for GVHD analysis were coded and read in a blinded fashion in-house.

### FMT.

Donor and recipient mice were both from National Cancer Institute/Charles River Laboratories facility. Donor mice used as a source of fecal pellets/dirty bedding (FMT) were purchased from Taconic’s or Jackson’s facilities. Dirty bedding and fecal materials from 1 cage of 5 mice were transferred to 2 recipient cages (5 mice/cage), and this was repeated 3 times at 2-day intervals for a week before BMT, followed weekly until 30 days after BMT.

### Gut permeability assay.

Food and water were withheld from all mice for 4 hours on day +7. FITC-dextran (MilliporeSigma) was administered by a 20-gauge 1.5-inch flexible intragastric gavage needle (Braintree Scientific) at a concentration of 800 mg/kg (approximately 16 mg/mouse). Four hours later, serum was collected from peripheral blood, diluted 1:1 with PBS, and analyzed on a plate reader (BioTek Instruments, catalog FLx800) at an excitation and emission wavelength of 485 nm and 535 nm, respectively. Concentrations of FITC-dextran in experimental samples were determined on the basis of serum from BM alone group or from naive mice.

### SCFA quantification.

To determine targeted fatty acid quantitation, we collected gut tissue samples from mice 21 days after BMT, homogenized them, and then snap-froze them in dry ice and kept them at –80°C. The frozen samples were shipped to Microbiome Insights Inc. for analysis (University of British Columbia, Vancouver, British Columbia, Canada) (64). Briefly, equal volumes of homogenized tissue were used. Samples were dispersed in acidified water spiked with stable isotope-labeled SCFA standards and extracted with diethyl ether, which was analyzed immediately by gas chromatography/mass spectrometry using a Phenomenex ZB-WAX column on an Agilent 6890 GC with a 5973MS detector. Quantitation was done by calibration to internal standards. The tissue levels were normalized by the protein concentration of the homogenized tissue.

### Quantitative PCR.

SI pieces (distal end of ileum) were snap-frozen, then ground using a mortar and pestle on dry ice. Total RNA was extracted using TRIzol reagent (Invitrogen, Thermo Fisher Scientific) according to the manufacturer’s recommendations. cDNA synthesis was performed using the Superscript first-strand cDNA synthesis system (Bio-Rad). Cytokine- and transcription factor–specific primer sets were used, and real-time quantitative PCR was performed using the SYBR Green method. The specific primer pairs were used as follows: ZO-1, 5′-CAACATACAGTGACGCTTCACA-3′ and 5′-CACTATTGACGTTTCCCCACTC-3′; Claudins1, 5′-GAAAGCTAGGTCGTGGGTCA-3′ and 5′-TCATAACTCCGGTCCCTCTG-3; JAM-C, 5′-GCTCCTGACAAAGCCACC-3′ and 5′-ATCACCCTAATCCCCATAAT-3′; S100A8, 5′-GGAAATCACCATGCCCTCT-3′ and 5′-TTTATCACCATCGCAAGGAAC-3′; GPR109A, 5′-GTTACAACTTCAGGTGGCACGAT-3′ and 5′-CTCCACACTAGTGCTTCGGTTATT-3′; GPR43, 5′-ACAGTGGAGGGGACCAAGAT-3′ and 5′-GGGGACTCTCTACTCGGTGA-3′; Reg3γ, 5′-TTCCTGTCCTCCATGATCAAAA-3′ and 5′-CATCCACCTCTGTTGGGTTCA-3′; and IL-22, 5′-GTGGAGAGATCAAGGCGATT-3′ and 5′-CAGACGCAAGCATTTCTCAG-3′.

### Examination of gut microbial communities.

Total DNA samples were prepared from SI (distal ileum) either using TRIzol reagent (Invitrogen, Thermo Fisher Scientific) (65) or using QIAamp PowerFecal DNA kit (QIAGEN) (66). Briefly (isolation of DNA using TRIzol reagents) ground intestinal samples were processed using TRIzol reagent as recommended by the manufacturer and centrifuged at 17,949*g*, and DNA was precipitated from the interphase and organic phase using 0.1 mol/L trisodium citrate in 10% ethanol. DNA was washed, dried, and suspended in 8 mmol/L NaOH, and the insoluble material was removed by centrifuging at 17,949*g*. Briefly (isolation of DNA using QIAamp PowerFecal DNA kit) fecal pellet or SI (distal ileum) was transferred to a Dry Bead Tube provided in the kit, and subsequent steps were performed according to the manufacturer’s instructions. The bead-beating step was performed with the Mini-BeadBeater-24 (BioSpec Products, Inc.). DNA was eluted in 100 μL C6 elution buffer solution. The extracted DNA samples were sent to a commercial sequencing facility (67) (MRDNA). Libraries were prepared following the Illumina TruSeq DNA library preparation protocol. Sequencing was performed using Illumina MiSeq platform with v3 300 base single read protocols.

### Statistics.

For comparison of recipient survival among groups in GVHD experiments, the log-rank test was used to determine statistical significance. Clinical scores and body weight loss were compared using a nonparametric Mann-Whitney *U* test. To compare pathology scores and cytokine levels, a 2-tailed Student’s *t* test was performed. A *P* value of less than 0.05 was considered significant. Statistical tests were performed in each experiment between control and *B*. *fragilis* treatment, and *P* values between these groups were indicated as shown. If statistical significance was not indicated, this means the relevant groups were compared but did not reach statistical significance (*P* > 0.05). Community-level differences in microbiome were analyzed with Bray-Curtis dissimilarities using robust multivariate Welch MANOVA test Wd* from Hamidi et al. 2019 (68). We applied univariate analyses to taxa that were present at more than 1% relative abundance in at least 1 sample. Relative abundances were compared using 1-way Kruskal-Wallis ANOVA with post hoc Mann-Whitney *U* tests, while centered log ratio–transformed abundances were compared using 1-way ANOVA and post hoc *t* tests, as indicated. Trends in alpha diversity analyses were determined using linear regression trend test. Multiple comparisons were adjusted for false discovery and considered significant for *q* < 0.1, which is consistent for discovery studies. All analyses were done in R statistical programming environment.

### Study approval.

The present studies in animals and xenograft models were reviewed and approved by an appropriate institutional review board. Experiments were carried out under protocols approved by the Institutional Animal Care and Use Committee of the MUSC in Charleston, South Carolina.

## Author contributions

MHS and XZY designed the study; MHS, YW, SS, DB, TT, HJC, LT, CM, and AVA participated in conducting experiments and acquiring or analyzing data. CL performed pathological analysis. MHS, CW, KEA, AVA, and XZY participated in interpreting data. MHS and XZY drafted and revised the manuscript. All authors proofread and edited the manuscript.

## Supplementary Material

Supplemental data

## Version 1 02/08/2021

Electronic publication
