# Supplementary material for: A single strain of Bacteroides fragilis protects gut integrity and reduces GVHD
Source: JCI Insight. 2021 Feb 8;6(3):e136841. doi: 10.1172/jci.insight.136841 (PMC7934839; doi:10.1172/jci.insight.136841)
Supplement: Supplemental data [file jciinsight-6-136841-s020.pdf]

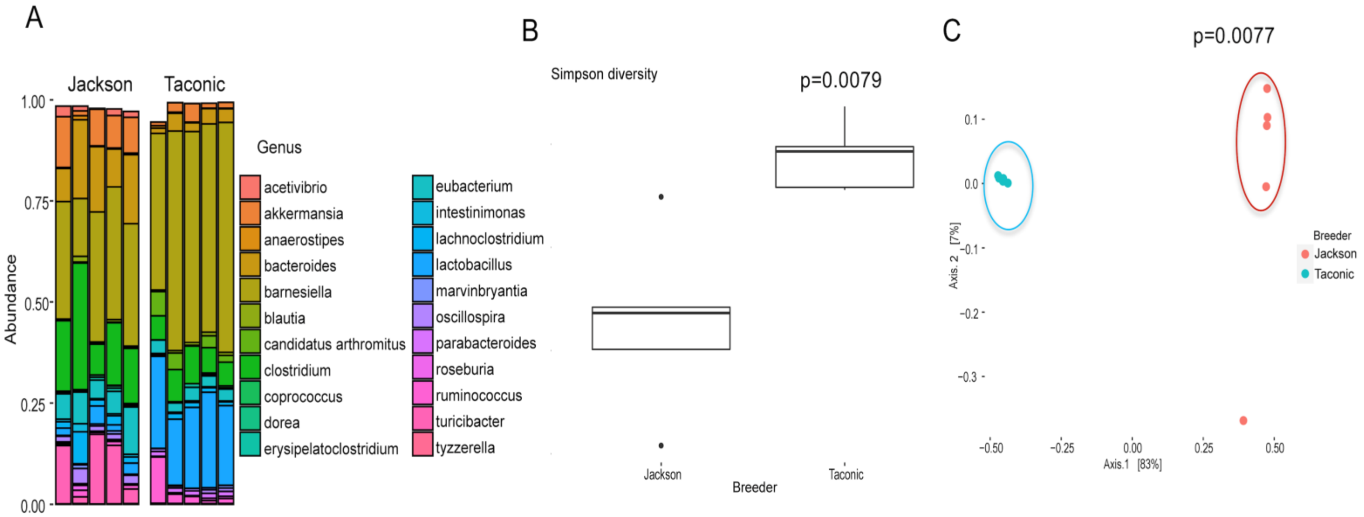

Figure S1: Gut microbiota in Taconic Farm versus Jackson Laboratory. Fecal pellets were collected from the Taconic (GT facility) and Jackson B6 mice. Total DNA was extracted for 16S rRNA sequencing. Bacterial composition is depicted at genus level (A), diversity is shown by Simpson diversity index (B), and Principal Coordinates Analysis (PCoA) is demonstrated (C). Data shown is from 5mice/group.

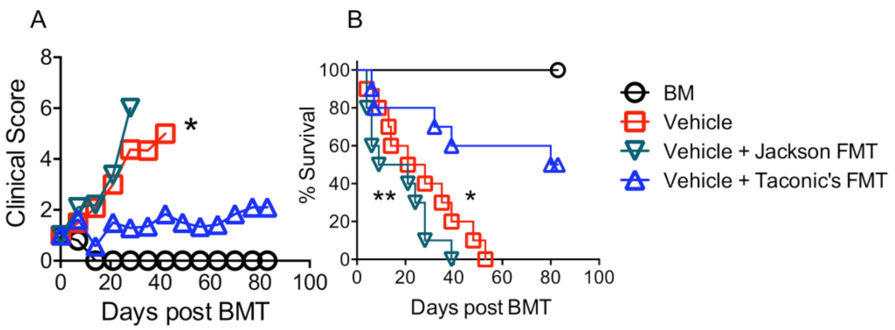

Figure S2: FMT from different donors impacts GVHD development. BALB/c mice were treated with busulfan at 20mg/kg and cyclophosphamide at 120 mg/kg daily for 6 days and then injected with 15×10^6/mouse TCD-BM alone or plus 15×10^6 total splenocytes from normal B6 mice. The bedding of recipient cages was replaced with clean bedding without (control), or with fecal pellets of B6 mice from either Jackson or Taconic. The FMT procedure was done thrice a week starting at two weeks prior to BMT and then weekly for a month post-BMT. Recipients were monitored for clinical score (A) and survival (B) (n=10 per group). Data shown was pooled from two replicate experiments. *p< .05,

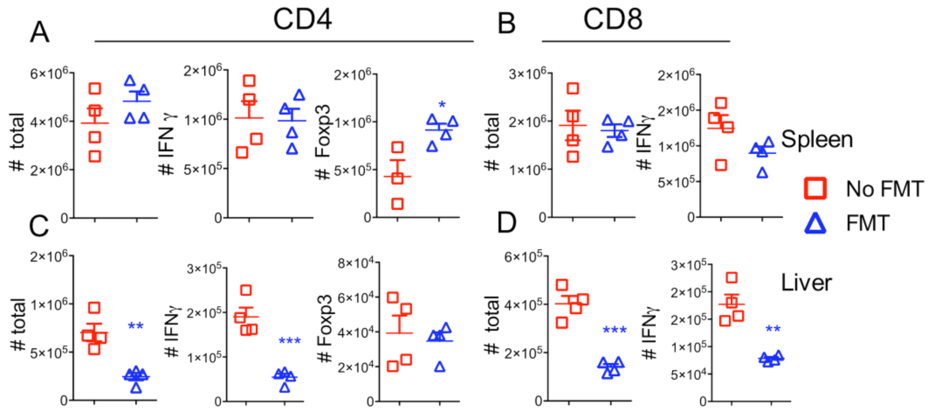

Figure S3: Effect of FMT on donor T-cell expansion and migration after allo-BMT. B6 → BALB/c BMT was initiated and FMT was administered as described in figure 2. Three weeks after BMT, spleens and liver were collected from the recipients and mononuclear cells were isolated and subjected to cell counting and FACS staining for surface H2K^{b} (donor MHC), CD4, CD8 and intracellular IFNγ and Foxp3. Absolute number of CD4 and CD8 were shown on gated live H2K^{b+} donor cells from spleen (A-B) and liver (C-D). Absolute numbers of IFNγ+ and Foxp3^{+} on CD4 (A-C) or CD8 donor cells (B-D) shown in recipient spleen and liver. Data represents one of 3-5 mice in each group of recipients. *p< .05, **p < .01, ***p < .001.

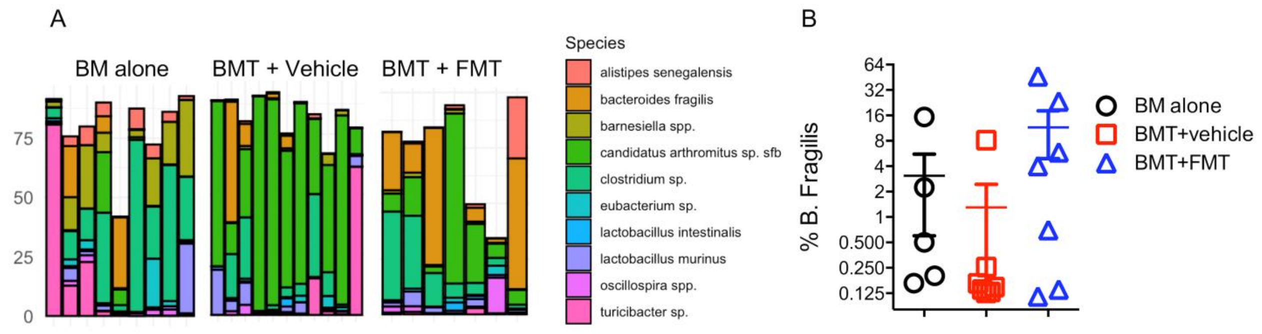

Figure S4: Abundance of *B. fragilis* increases after FMT treatment: B6 $\rightarrow$ BALB/c BMT was initiated and FMT was administered.The bedding of recipient cages was replaced by bedding without (control), or with fecal pellets from Taconic B6 mice. The FMT procedure was done thrice a week starting at 2 weeks prior to BMT and then weekly after BMT. Three weeks after BMT, ileums were collected from the recipients and extracted for total DNA, which was used for 16S rRNA sequencing. Bacterial composition at the species level is depicted (A) and percentage of *B. fragilis* is shown as an average of two combined group (n=7-10).

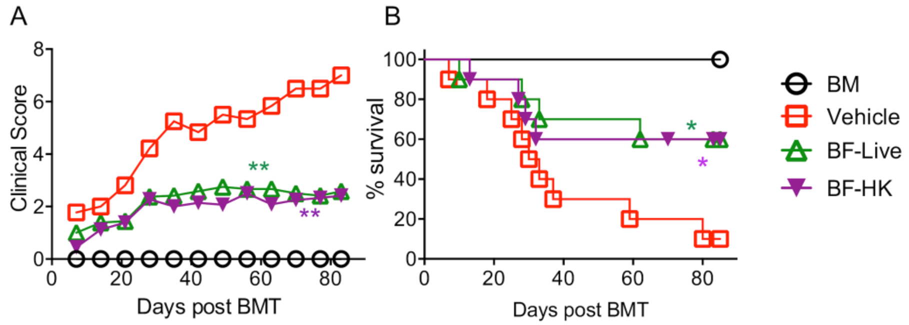

Figure S5: *Live or heat killed B. fragilis* ameliorates GVHD*.* BALB/c mice were pre-conditioned as described in figure 3. The recipients were administered thrice a week through oral gavage with live ~10^9 *B.fragilis* colony forming unit or heat-killed *B. fragilis*, starting two weeks before BMT and then weekly until up to a month after BMT. Recipients were monitored for clinical score (A) and survival (B) until 80 days post- BMT (n=10 per group). Data shown was pooled from two replicate experiments. Asterisks indicate statistical significance *p< .05, **p < .01.

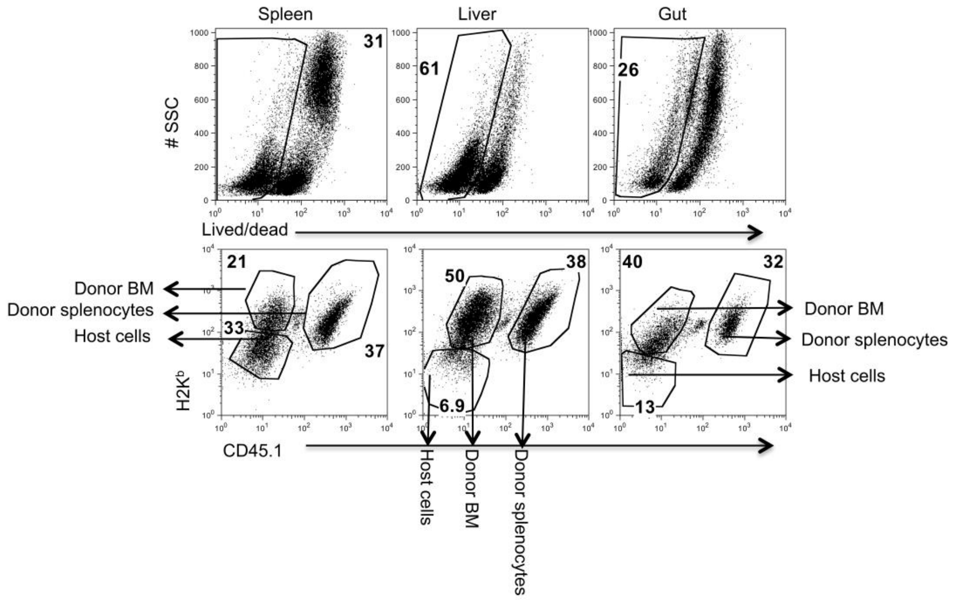

Figure S6: Gating strategy on donor and recipient cells. Cells were stained for live dead markers. Among live cells, H2K^{b+} cells are derived from donor BM and H2K^{b+}CD45.1$^+$ cells are from donor splenocytes. Donor splenocytes were gated for further analysis on various surface and intracellular markers.

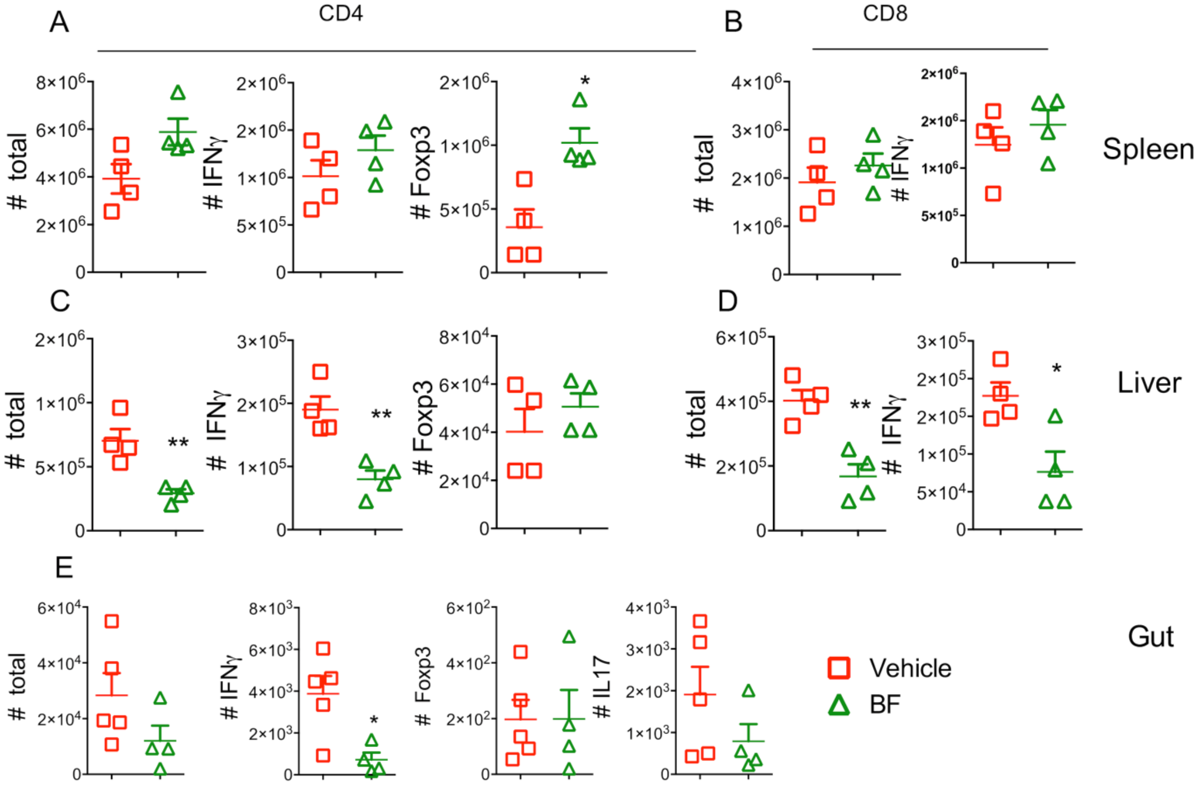

Figure S7: Effect of *B. fragilis* on donor T-cell expansion and migration after allo-BMT. Experiments were performed as described in figure 4. Three weeks after BMT, spleens and liver were collected from the recipients and mononuclear cells were isolated and subjected to cell counting and FACS staining for surface $H2K^b$ (donor MHC), CD4, CD8 and intracellular IFNγ and Foxp3. Absolute number of CD4 and CD8 shown on gated live $H2K^{b+}$ donor cells from spleen (A-B), liver (C-D), and gut (CD4) (E), respectively. Absolute numbers of $IFN\gamma^+$ and $Foxp3^+$ cells among donor CD4 (A-C) or CD8 cells (B-D) and absolute numbers of $IFN\gamma^+$ IL17 and $Foxp3^+$ cells among donor CD4 (E) were shown in recipient spleen, liver and gut . Data are from one of two representative experiments. Asterisks indicate statistical significance *p< .05, **p < .01,

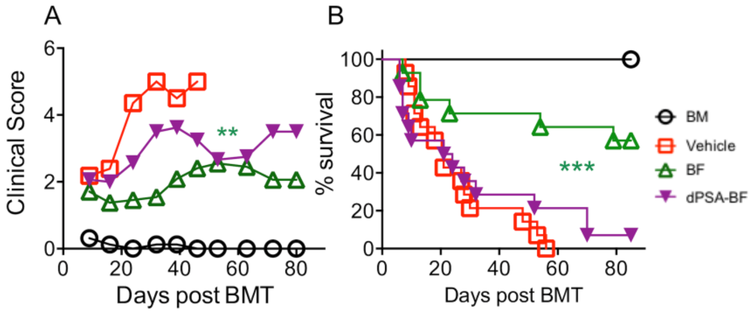

Figure S8: Requirement of PSA for *B. fragilis* protection against GVHD production*.* BALB/c mice were pre-conditioned and transplanted as described in figure 3. The recipients were administered thrice a week through oral gavage with live ~10^9 *B. fragilis* colony forming unit (WT BF or ∆PSA BF), starting 2 weeks before BMT and then weekly until up to a month after BMT. Recipients were monitored for clinical score (A) and survival (B) until 80 days post-BMT (n=10 per group). Data shown was pooled from two replicate experiments. Asterisks indicate statistical significance $**p < .01$, $***p < .001$,
